# Risk of second primary cancers in cancer patients treated with cisplatin: a systematic review and meta-analysis of randomized studies

**DOI:** 10.1186/s12885-017-3902-4

**Published:** 2017-12-19

**Authors:** Fei Liang, Sheng Zhang, Hongxi Xue, Qiang Chen

**Affiliations:** 1 Shanghai Cancer Center and Shanghai Medical College, Fudan University, Shanghai, China; 2 Rizhao City Hospital of Traditional Chinese Medicine, 35 Wanghai Road, Rizhao, China; 3 Department of clinical biochemistry, School of public health Taishan medical university, Taishan, China; 4 Medical Oncology, Shanghai Cancer Center, Fudan University, 270 Dongan Road, Shanghai, 200032 China

**Keywords:** Second cancer, Cisplatin, Randomized controlled trials

## Abstract

**Background:**

Case reports, retrospective analyses, and observational studies have linked the use of cisplatin to increased risk of second cancers, especially life-threatening secondary leukemia. We therefore performed a systematic review and meta-analysis to evaluate the risk of second cancers associated with receipt of cisplatin-based chemotherapy in randomized controlled trials (RCTs).

**Methods:**

We searched MEDLINE, Embase, the Cochrane Central Register of Controlled Trials, trial registers, conference proceedings, review articles, and reference lists of trial publications for all relevant RCTs comparing cisplatin- versus non-cisplatin-containing chemotherapy with data on second cancers. We extracted data about study characteristics and second cancers, especially leukemia/ myelodysplasia. The primary and secondary outcomes were the odds ratios (ORs) for all second cancers and for secondary leukemia/ myelodysplasia, respectively.

**Results:**

We identified 28 eligible trials with 7403 patients. Second cancers were reported in 143 patients, including 75 patients in the cisplatin arm and 68 in the non-cisplatin arm (raw event rates of 1.91 and 1.96%, respectively). The pooled OR for risk of all second cancers associated with cisplatin-based chemotherapy was 0.95 (95% confidence interval (CI): 0.67–1.33, *P* = 0.76). Secondary leukemia/ myelodysplasia was reported in 14 patients on cisplatin arms and in 6 patients on non-cisplatin arms of 11 eligible RCTs with 2629 patients (raw event rates of 1.09 and 0.45%, respectively; pooled OR = 2.34, 95%CI 0.97–5.65, *P* = 0.06).

**Conclusion:**

Cisplatin was not associated with a significantly increased risk of second cancers compared with non-cisplatin-based chemotherapy. There is a non-significant trend to increased risk of leukemia/ myelodysplasia and the absolute risk was low. The concern about risk of second cancers should not influence decisions to use an efficacious regimen containing cisplatin.

**Electronic supplementary material:**

The online version of this article (10.1186/s12885-017-3902-4) contains supplementary material, which is available to authorized users.

## Background

Second primary cancers in cancer survivors now constitute 18% of all cancer diagnoses in the US Surveillance, Epidemiology and End Results (SEER) cancer registries [1, 2]. In addition to this high morbidity, second cancers also lead to substantial mortality. For example, second primary cancers have become the leading cause of mortality among patients with Hodgkin’s lymphoma [1, 3, 4]. Thus, elucidation of factors leading to a second cancer and methods to avoid it may have substantial impact on both individual patient outcomes and public health. The increased risk of developing second cancers among cancer survivors is probably due to a combination of life-style, genetic factors, and treatment for the first cancer such as radiotherapy and certain chemotherapy regimens [3–5].

Since its introduction into clinical practice in 1970s, cisplatin, a chemotherapeutic agent binding to and causing crosslinking of DNA, has quickly become the cornerstone of modern chemotherapeutic treatment and been widely used worldwide because of its efficacies against various malignancies [6]. However, the persistence of platinum-DNA adducts in numerous human tissues long after treatment has completed has led to concerns that cisplatin-based chemotherapy might be associated with a greater risk of second cancers than other types of chemotherapy [6]. Indeed, it has been documented that cisplatin can be carcinogenic both in laboratory animals and humans [7–9]. Recently, using the population-based SEER cohort, Fung et al. found that cancer patients treated with cisplatin-based chemotherapy had a 40% increased risk of developing secondary solid cancers after initial diagnosis when they were compared with patients treated with surgery alone [10, 11]. In a large case-control study of patients with testicular cancer, the estimated relative risk of leukemia was 3.2 (95% confidence interval [CI] = 1.5–8.4) when cisplatin was given [12]. Another large case-control study of patients with ovarian cancer documented that the relative risks of leukemia was 3.3 (95% CI = 1.1–9.4) for cisplatin treatment [13]. Moreover, strong dose-response relationships between cumulative cisplatin dose and secondary leukemia risk (*p* < .001) were demonstrated in both studies [12, 13]. These studies have seriously raised the concern about possible second cancer risk with the use of cisplatin. However, these studies were limited by their retrospective or observational design. In fact, there is no level-1 evidence showing an increased risk of second cancer associated with cisplatin-based chemotherapy in the context of the substantial number of patients that received cisplatin worldwide every year. Therefore, we performed an up-to-date systematic review and meta-analysis to evaluate the effect of cisplatin on risk of second cancer in patients treated for their first cancer in RCTs with arms that compared chemotherapy regimens that did and did not include cisplatin.

## Methods

### Selection criteria and search strategy

The selection and systematic review of trials was performed in accordance with the Preferred Reporting Items for Systematic Review and Meta-Analyses (PRISMA) statement [14].

We searched Medline, Embase, and the Cochrane Central Register of Controlled Trials (CENTRAL) from inception to 24 March 2016. We combined both MeSH and free text words to identify relevant studies. The search strategy (Additional file 1) was developed based on an existing search strategy. ClinicalTrials.gov was also searched in June 2016 to ensure data from previously published trials were updated. We limited our search to “interventional” trials with available results. Conference Proceedings from the American Society of Clinical Oncology and the European Society for Medical Oncology for the years 2010 to 2015 were also hand searched. Finally, reference of all eligible studies was also hand searched for other relevant citations.

Eligible studies were trials in which cancer patients were randomly assigned to treatment with cisplatin- versus non-cisplatin-containing chemotherapy. Studies that compared chemotherapy with radiotherapy, targeted therapy, surgery or placebo were excluded. In addition, eligible studies were required to report the incidence of second cancers in each treatment arm. Both the text and supplements of reports were screened to identify whether data on second cancers were available.

We used the Cochrane Collaboration’s tool to assess the risk of bias of RCTs included in our study [15]. Random sequence generation, allocation concealment, blinding of participants, personnel, and assessors of outcome, incomplete outcome data and selective outcome reporting were judged to be of low, unclear, or high risk for each trial. We assessed potential publication bias by visual inspection of the symmetry of funnel plots and with the Begg and Egger tests.

### Data extraction

For all eligible trials, we extracted the following data: trial phase, year of publication, underlying malignancy, length of follow-up, median age, adjuvant/metastatic setting, chemotherapy regimens used in each treatment arm, actual accumulative total cisplatin dose(mg/m^2^) (if not available, planned total dose was used), number of patients enrolled, the number and cancer types of all second cancers in each treatment arm. If insufficient data regarding second cancers were retrieved from publications, we sought it by contacting the corresponding authors.

For multiple reports of the same trial, we combined all data. Only data from the longest follow-up time was used when data was reported at multiple follow up periods.

Two authors (S.Z and F.L) independently screened trials for eligibility, assessed risk of bias and extracted required data from each included trials using standardized forms. Any discrepancy was identified and resolved successfully by consensus of all authors. Cronbach’s alpha was 0.8.

### Outcomes

The primary outcome of this analysis is the odds ratios (OR) of second cancers associated with cisplatin- versus non-cisplatin-based chemotherapy. OR > 1 means second cancers are more likely to occur in the cisplatin arm than in the non-cisplatin arm. The secondary outcome is the OR of second leukemia/myelodysplasia.

### Statistical analysis

Meta-analysis was performed with Review Manger 5.3 (Nordic Cochrane Centre, Cochrane Collaboration, 2014).

Many trials had few second cancers and the event rates were low, so the odds ratios and 95% confidence intervals were calculated with the use of the Peto method [15, 16]. Trials in which patients had no events in both cisplatin and non-cisplatin arms were excluded from meta-analyses. Heterogeneities were assessed using χ2 test and the I^2^ statistic. A two-tailed *P* value of less than 0.05 was considered as statistically significant.

To better understand the relationship between cisplatin and second cancers, we performed six pre-specified subgroup analyses stratifying patients by type of control in trials evaluating cisplatin versus another platinum agent or non-platinum chemotherapy (non-platinum control chemotherapy vs other platinum-based control chemotherapy) [17]; length of follow-up (≤ 60 vs > 60 months); total cisplatin dose (≤ 300 vs > 300 mg/m^2^), mode of treatment (chemotherapy alone vs chemotherapy and radiotherapy), mode of comparison (confounded vs un-confounded),and setting (adjuvant vs metastasis).The designation of the cut-points of both length of follow and accumulative total cisplatin dose was based on previous studies [12, 13]. Comparison of cisplatin arm and control arm were classified into three categories: cisplatin ± other therapy regimen vs other cytotoxic drug ± the same therapy regimen (eg. cisplatin vs carboplatin or doxorubicin and cisplatin vs doxorubicin and paclitaxel); cisplatin plus other chemotherapy regimens vs the same chemotherapy regimen without cisplatin(eg, epirubicin and cisplatin vs epirubicin); cisplatin plus other chemotherapy regimen vs different chemotherapy regimen (eg. cisplatin, doxorubicin and cyclophosphamide vs chlorambucil). The first two groups were considered to be un-confounded comparison [17–19].

Given concerns that Peto methods may not be ideal for evaluation of rare events with baseline event rate above 1%, we also carried out sensitivity analyses using Mantel-Hanszel methods. We also conducted two extra sensitivity analyses by using alternative effect measure (odds ratio vs relative risk) and statistical models regarding heterogeneity (fixed vs random effects) to further assess the robustness of the results to the choice of this model for the meta-analysis.

## Results

### Search results

**Fig. 1:**
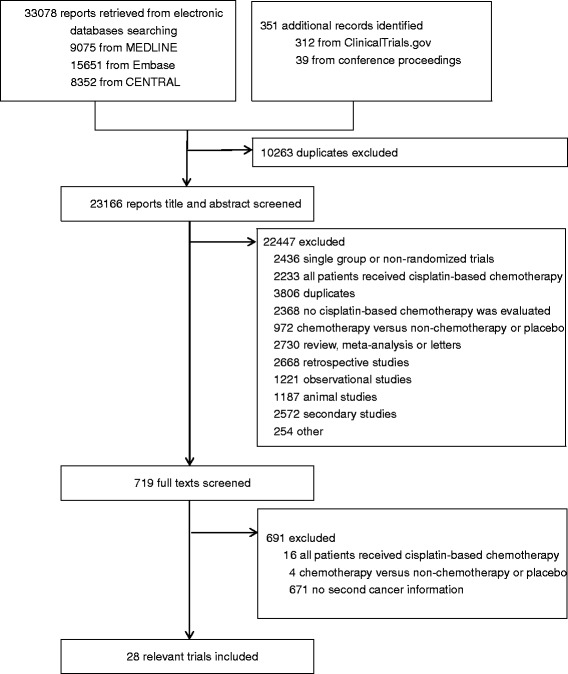
Study flow chart. CENTRAL = Cochrane Central Register of Controlled Trials

Our initial search yielded 33,429 records. After removing obvious duplicates and screening titles and abstracts, we retrieved 719 reports for full text screening. Twenty-eight studies (27 from journals and one from conference abstract) were eligible for inclusion (Fig. 1) [20–47]. Two studies included multiple cisplatin arms, which were combined for this analysis. Of the 28 RCTs, 2 trials reported no incidence of any second cancers in both cisplatin and non-cisplatin arms, 15 trials reported the detailed information of cancer types of second cancers (10 trials reported incidence of secondary leukemia/myelodysplasia only), 11 trials did not provide detailed information of types of second cancers (Additional file 1: Table S1).

### Risk of bias

None of the included trials was placebo controlled or double blinded, which would be difficult given the hydration and antiemetic therapy necessary with cisplatin-based chemotherapy. Most trials adequately generated their randomization sequence and concealed allocation, and the risk of incomplete and selective reporting of outcomes was assessed to be low. (Additional file 1: Table S2).

### Publication bias

No evidence for publication bias was demonstrated based on the assessment of the funnel plot or formal analysis (Begg test, *P* = 0.96; Egger test, *P* = 0.80).

### Study, patient, and treatment characteristics

A total of 7403 patients from 28 RCTs were included. The Characteristics of each trial are summarized in Table 1. The trials were performed in patients with head and neck cancer (6 trials), ovarian cancer (6 trials), and other multiple types of cancer (16 trials). The control chemotherapy regimens consisted of other platinum-based therapy in eight trials (carboplatin, 7; oxaliplatin, 1) and non-platinum-based therapy in 20 trials. The leading underlying malignancies were head and neck cancers (6) and ovarian cancers (6). Accumulative total cisplatin dose was available for 27 trials, ranging from 64 to 580 mg/m^2^. Follow up time were reported or estimable in all trials, ranging from 17 to 156 months. Nine trials involved radiotherapy in both cisplatin and non-cisplatin arms. Mode of comparison was classified as un-confounded in 13 trials and confounded in 16 trials with one study included both confounded and un-confounded comparisons.

**Table 1:** Characteristics of randomized controlled trials included in the meta-analysis

Reference	Year of publication	Trial Phase	Cancer Type	Follow-up (month)	Total cisplatin dose(mg/m^2^)	Cisplatin Arm				Non-cisplatin Arm			
						Regimen	No. of Patients	Second Cancers (No.)	Leukemia (No.)	Regimen	No. of Patients	Second Cancers (No.)	Leukemia (No.)
Ajani et al..	2008	III	Anal	Median 30.1	Median 300	Fluorouracil, cisplatin and radiotherapy	320	1		Fluorouracil, mitomycin and radiotherapy	324	0	
Basu et al	2016	II	Cervical	Median 29.2	Median 200	Cisplatin and radiotherapy	105	0		Interferon-alpha, retinoic acid and radiotherapy	104	1	
Booton et al	2006	III	Lung	Median 17.4	Median 200	Mitomycin, ifosfamide and cisplatin or mitomycin, vinblastine and cisplatin	210	0		Docetaxel and carboplatin	212	1	
Cohen et al	2014	III	Head and neck	Minimum 30	Median 150	Docetaxel, cisplatin and fluorouracil, followed by docetaxel, fluorouracil, and hydroxyurea plus radiotherapy	138	2		Docetaxel, fluorouracil, and hydroxyurea plus radiotherapy	135	1	
Conroy et al	2014	II/III	Oesophageal	Median 25.3	Median 300	Fluorouracil, cisplatin and radiotherapy	133	7		Fluorouracil, leucovorin, oxaliplatin and radiotherapy	134	8	
Cortelazzo et al	2012	III	Lymphoma	Median 27.7	Planned 300	Rituximab, cyclophosphamide, Ara-C, methotrexate, etoposide, cisplatin and autografting	121	0		Rituximab, cyclophosphamide, doxorubicin, vincristine and prednisone	127	0	
du Bois et al	2003	III	Ovarian	Mean: 48.5 for cisplatin arm 49.9 for non-cisplatin arm	Median 441.6	Paclitaxel and cisplatin	386	9	0	Paclitaxel and carboplatin	397	9	0
Fleming et al	2004	III	Endometrial	Median 61 for alive patients	Planned 350	Doxorubicin and cisplatin	156	0	0	Doxorubicin and paclitaxel	157	1	1
Fountzilas et al	2004	III	Head and neck	Median 60	Median 270	Cisplatin and radiotherapy	45	1		Carboplatin and radiotherapy	38	1	
Garden et al	2004	II	Head and neck	Median: 31.2–34.8	Median 100/140	Cisplatin, fluorouracil and radiotherapy vs cisplatin, paclitaxel and radiotherapy	155	6		Hydroxyurea, fluorouracil and radiotherapy	76	1	
Geyer et al	2004	II	Brain	Median 79.2	Not reported	Vincristine, cisplatin, cyclophosphamide and etoposide	149	3	3	Vincristine, carboplatin, ifosfamide and etoposide	135	1	1
Harari et al	2014	II	Head and neck	Median 52.8	Median 180	Cisplatin, cetuximab and radiotherapy	97	4		Docetaxel, cetuximab and radiotherapy	106	12	
Hiesiger et al	1995	Not reported	Brain	Median 26.5 for alive patients	Planned 720	Cisplatin	125	0	0	PCNU	145	1	1
Homma et al	2004	II	Head and neck	Median 63	Median 64	Cisplatin and radiotherapy	59	7		Carboplatin and radiotherapy	60	7	
Intragumtornchai et al	2000	Not reported	Lymphoma	Median 36	Maximum 400	Etoposide, methylprednisolone, high-dosecytarabine, cisplatin, high-dose therapy and autologous peripheral blood progenitor cell transplantation	23	1	1	Cyclophosphamide,doxorubicin, vincristine and prednisolone	25	0	0
James et al	2013	III	Anus	Median 61.2	Planned 120/120/240	Cisplatin, fluorouracil and radiotherapy vs Mitomycin, fluorouracil, radiotherapy followed by cisplatin and fluorouracil vs Cisplatin, fluorouracil, radiotherapy followed by cisplatin and fluorouracil	694	17		Mitomycin, fluorouracil and radiotherapy	246	3	
Jennings et al	2002	II	Glioma	Maximum 37	Median 300	Cisplatin, etoposide, cyclophosphamide and vincristine	31	0	0	Carboplatin, etoposide, and vincristine	32	1	1
Nicoletto et al	2007	Not reported	Ovarian	Median 178	Median 375	Cisplatin and cyclophosphamide	80	0		Adriamycin and cyclophosphamide	81	2	
Nielsen et al	2000	III	Breast	Mean 17	Median 518	Epirubicin and cisplatin	74	3	3	Epirubicin	81	0	0
Olasz et al	2004	Not reported	Head and neck	Median 52	Median 120	Bleomycin, vincristine, methotrexate and cisplatin	19	1		Bleomycin, vincristine and methotrexate	19	1	
Sirohi et al	2010	III	Breast	Median 112	Median 360	Epirubicin, cisplatin and 5-fluorouracil	172	3		5-fluorouracil, epirubicin and cyclophosphamide	177	11	
Sutton et al	2000	III	Uterus	Maximum 24	Median 320	Ifosfamide and cisplatin	90	1	1	Ifosfamide	101	0	0
Taylor et al	1994	III	Ovarian	Median 108	Median 331.2	Cisplatin	64	0		Carboplatin	67	0	
Tropé et al	1996	Not reported	Ovarian	Minimum 120	Planned 500	Cisplatin, doxorubicin and melphalan	143	4	4	Doxorubicin and melphalan	153	2	2
Tsimberidou et al	2002	Not reported	Lymphoma	Median 70.8	Planned 100	CHOD-Bleo, ESHAP, and NOPP	69	1	1	Fludarabine, mitoxantrone, and dexamethasone	1	73	1
Wada et al	1996	Not reported	Lung	Median 60	Mean 56.5	Cisplatin, vindesine, tegafur and uracil	109	3		Tegafur and uracil	103	2	
Wadler et al	1996	III	Ovarian	Median 156 for alive patients	Median 580	Cyclophosphamid, hexamethylmelamine, doxorubicin, and cisplatin	126	0		Melphalan	118	1	
Williams et al	1985	Not reported	Ovarian	Median 45	Planned 400	Cisplatin, doxorubicin, cyclophosphamide	40	1	1	Chlorambucil	44	0	0

*CHOD-Bleo* cyclophosphamide, Doxorubicin, Vincristine, Bleomycin and Dexamethasone. *ESHAP* Etoposide, Methylprednisolone, Cytarabine and Cisplatin. *NOPP* Mitoxantrone, Vincristine, Procarbazine and Prednisone

### Second cancers

**Fig. 2:**
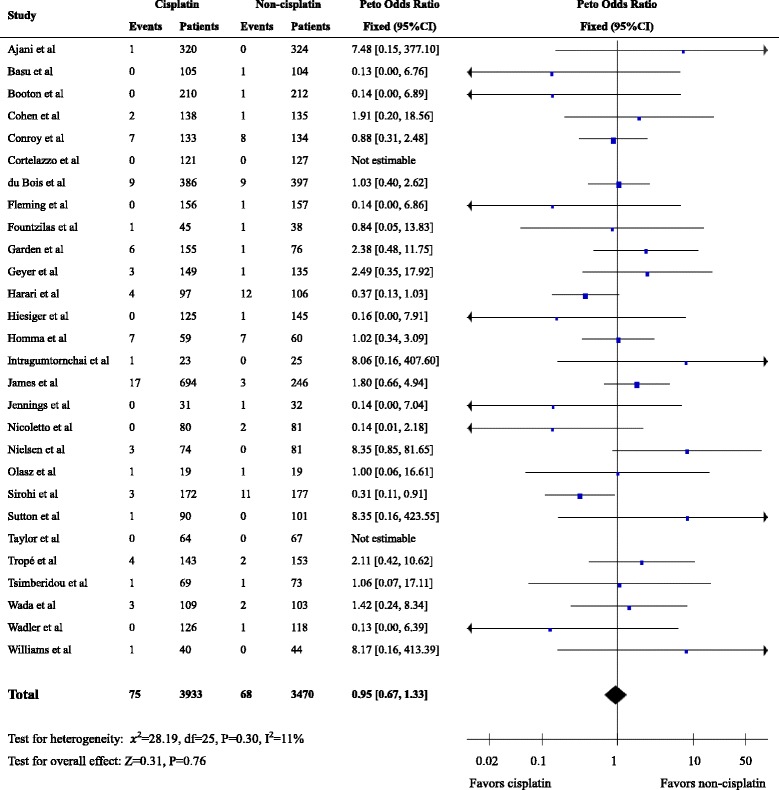
Forest plot of the odds ratio of second cancers associated with cisplatin- versus non-cisplatin-based chemotherapy

Second cancers were reported in 143 patients, including 75 patients in the cisplatin arm (raw event rate 1.91%) and 68 in the non-cisplatin arm (raw event rate 1.96%). The incidence rate of second cancers varied among trials, ranging from 0 to 11.9%. The highest incidence of second cancers was observed in a trial of 119 patients with head and neck cancers [39]. The estimated OR of second cancers for cisplatin-versus non-cisplatin-based chemotherapy was 0.95 (95% CI, 0.67–1.33, *P* = 0.76) (Fig. 2).

**Fig. 3:**
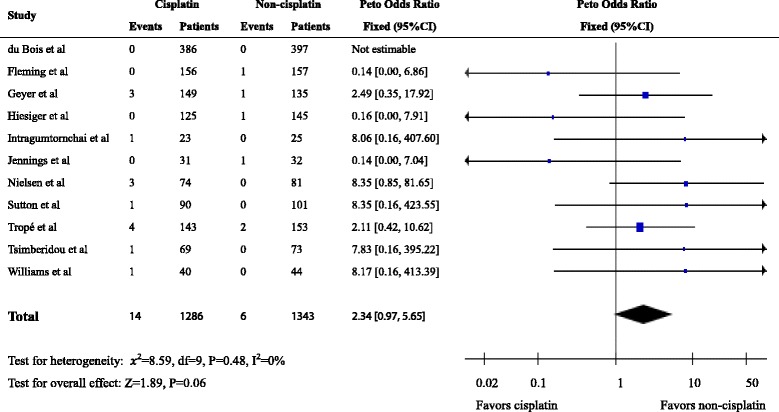
Forest plot of the odds ratio of leukemia associated with cisplatin- versus non-cisplatin-based chemotherapy

Secondary leukemia/myelodysplasia was reported in 11 eligible RCTs representing 2629 patients, whereas 17 studies did not report leukemia. It is unclear from the publications whether this reflects the absence of leukemia/myelodysplasia in these 17 studies or a failure to report this specific type of second cancers. Leukemia/myelodysplasia was reported in 14 patients on cisplatin arm (raw event rate, 1.09%,) and in 6 patients on non-cisplatin arm (raw event rate, 0.45%). The pooled OR was 2.34 (95% CI, 0.97–5.65, *P* = 0.06) (Fig. 3).

### Subgroup analyses

To explore whether the possible increased risk of second malignancy was common to all platinum agents or unique to cisplatin, the control arm was further classified as platinum control or non-platinum control, according to whether other platinum agent such as oxaplatin or carboplatin was included. The subgroup analysis showed that cisplatin did not increase risk of second cancers compared with another platinum (OR 0.97, 95 CI 0.56–1.66) or non-platinum agents (OR 0.94, 95 CI 0.60–1.45) (Additional file 1: Figure S1). Neither high dose (OR 0.88, 95 CI 0.50–1.56) nor low dose (OR 0.94, 95 CI 0.61–1.46) was associated with increased risk of second cancers (Additional file 1: Figure S2). There was no significant relationship between the length of follow-up time and the pooled OR of second cancers(0.99 in trials with follow up time ≤ 60 months vs 0.89 in those > 60 months; interaction *P* = 0.77) (Additional file 1: Figure S3). We also classified trials into chemotherapy alone or chemotherapy and radiotherapy. No significant interaction effect was identified between these subgroups (0.90 vs 0.99; interaction *P* = 0.78) (Additional file 1: Figure S4). In some trials there were treatment regimen difference aside from cisplatin (eg, mitomycin, ifosfamide and cisplatin versus docetaxel and carboplatin). To address whether carcinogenicity of other regimen components would influence the results, we categorized trials into confounded or un-confounded according to the difference between treatment arms aside from cisplatin. There was no significant difference between the two subgroups (1.21 for confounded vs 0.88 for un-confounded; interaction *P* = 0.40) (Additional file 1: Figure S5) (Table 2). There was no significant difference (interaction *P* = 0.50) in the odds ratio of second cancers between cisplatin used in the adjuvant setting (Peto odds ratio = 0.90; 95% CI, 0.62–1.31)and metastasis setting(Peto odds ratio = 1.18; 95% CI, 0.59–2.37) (Table 2).

**Table 2:** Subgroup analysis of odds ratio (OR) of second cancers associated with cisplatin chemotherapy

Group	No. of trials	Cisplatin		Non-cisplatin		I^2^(%)	OR (95%CI)	P	
		No. of events	No. of patients	No. of events	No. of patients			OR	Interaction
Type of control									0.93
Non-platinum	20	48	2856	40	2395	29	0.94 [0.60, 1.45]	0.77	
Other platinum	8	27	1077	28	1075	0	0.97 [0.56, 1.66]	0.90	
Total cisplatin dose(mg/m^2^)									0.85
≤ 300	16	50	2461	41	1946	0	0.94 [0.61, 1.46]	0.80	
> 300	11	22	1323	26	1389	43	0.88 [0.50, 1.56]	0.66	
Follow-up time(months)									0.77
≤ 60	18	40	2221	39	2203	4	0.99 [0.63, 1.56]	0.97	
> 60	10	35	1712	29	1267	30	0.89 [0.53, 1.50]	0.67	
Mode of treatment									0.78
Chemotherapy alone	19	30	2187	34	2247	19	0.90 [0.55, 1.48]	0.68	
Chemotherapy and radiotherapy	9	45	1746	34	1223	5	0.99 [0.62, 1.59]	0.98	
Mode of comparison									0.40
Un-confounded	16	53	2602	51	2228	28	0.88 [0.59, 1.32]	0.54	
Confounded	13	22	1331	18	1318	0	1.21 [0.65, 2.28]	0.54	
Treatment setting									0.50
Adjuvant	20	61	2747	56	2252	0	0.90 [0.62, 1.31]	0.65	
Metastasis	8	14	1186	12	1218	0	1.18 [0.59, 2.37]	0.58	

### Sensitivity analysis

The sensitivity analysis using alternative effect measure (odds ratio vs relative risk), pooling method (Peto vs Mantel-Hanszel), and statistical models regarding heterogeneity (fixed vs random model) did not show any important change in the pooled OR for both second cancers and leukemia/myelodysplasia (Additional file 1: Figures S6, S7, S8, S9, S10 and S11).

## Discussion

Second primary cancer has become a substantial cause of morbidity and mortality in cancer survivors [1, 4]. Cisplatin-based chemotherapy can lead to cure or long-term remission in several types of cancer including testicular and ovarian cancer. Evaluation of long-term risk of second cancers due to cisplatin-based chemotherapy has become increasingly important in the context of the large number of patients receiving cisplatin worldwide each year [6]. We sought to comprehensively examine the relationship between cisplatin-based chemotherapy and risk of second cancer in patients with first cancer. Although a small number of patients with second cancers have been reported in RCTs of cisplatin-based chemotherapy, none of these trials were designed to have enough power to assess any potential risk of second cancer. Given the clinical significance of this topic, we pooled data from RCTs for further analysis. Indeed, our meta-analysis of 28 trials demonstrated that no increased risk of second cancer was associated with cisplatin-based chemotherapy versus those receiving non-cisplatin-based chemotherapy. This result is in contrast to previous reports from retrospective and observational studies [10–13], which were limited by selection bias and various known and unknown confounders. Randomized allocation of participants could avoid such biases. The result of meta-analysis of relevant RCTs represents the least biased evidence base in this regard. Other strengths of our study include the comprehensive search, careful selection of studies from published and non-published trials through various data sources.

Because only about half of the included studies provided detailed cancer types of second cancers, a further analysis of any types of second cancers cannot be performed. However, previous case-control studies have found possible association between cisplatin and second leukemia/myelodysplasia and documented a strong dose-response relationship [12, 13]. So we also explored the possible risk of second leukemia. We found a non-significant trend to increased risk of secondary leukemia (*p* = 0.06) in analysis of 11 studies with available data. It is noteworthy that the events are very low (16 in cisplatin arm versus 6 in non-cisplatin arm) and the confidence interval was wide. Due to the rarity of leukemia events, further studies to clarify this question may not be feasible. The length of follow-up duration has been well established to be associated with the incidence of second cancers [4, 48]. The relatively short follow-up time of the RCTs included in our trials, with 18 of the 28 included trials reported a median follow-up time no more than 5 years, may contribute to the low incidence rate of second cancers. However, the determination of risk of second cancers associated with cisplatin- versus non-cisplatin-based chemotherapy, the primary endpoint of our study, should not be affected, because the design of most RCTs should provide for relatively balanced, if not equal, follow-up of patients in both study arms for the duration of observation. Thus, current study represents the largest study with available information. Although we cannot completely exclude the possibility that a statistically significant increase in relative risk was missed due to the few events in RCTs included and borderline significance of results of secondary leukemia, the rarity of events suggests that such a finding would be very unlikely to change current benefit-risk balance of cisplatin-based chemotherapy in clinical practice.

We also performed subgroup analyses to better understand the relationship between cisplatin and second cancer. Diverse chemotherapeutic reagents have been used. In some trials, there were treatment regimen differences aside from cisplatin. In this case, we cannot rule out the possible contribution to risk of second cancers by other regimen components. The subgroup analyses about un-confounded and confounded groups did not show any difference between these subgroups. Because radiotherapy was used in both treatment arms in 9 studies and radiotherapy is an established risk factor for increased second cancer in previous studies, we also performed subgroup analysis comparing the radiotherapy-involved studies versus radiotherapy-not-involved studies. Indeed, although higher incidence rate of second cancers was observed in the patients receiving radiotherapy and chemotherapy combination, no differences were found between these radiotherapy-involved or radiotherapy-not-involved subgroups. Other subgroup analyses regarding the total dose of cisplatin, follow-up time, control chemotherapy reagents consistently showed there were not differences among these subgroups.

Notably, the total cumulative cisplatin doses in included studies were much lower than that previously reported in observational studies in which significant association of secondary leukemia with cisplatin was demonstrated [8, 9, 12, 13, 49], with majority of included trials reported total dose less than 300 mg/m^2^ and only one trial more than 600 mg/m^2^. In previous studies, total cisplatin dose at higher than 750 mg/m^2^ was associated with significantly increased risk of leukemia [12]. Modern chemotherapy regimens generally contain lower dose of cisplatin as reflected in the included RCTs, and this may contribute to the relatively low risk of second cancers or leukemia/myelodysplasia.

We should acknowledge other limitations in our study. The types of primary cancer in included trials are diverse, while previous conclusion about increased risk of second cancer associated with cisplation was drawn from studes in testicular cancer patients. Especially, the design of comparison of cisplatin versus non-cisplatin treatment arms may not be suited for investigation of germ cell tumors, where cisplatin has been the dominant and possibly the most significant chemotherapeutic. It is almost impossible to have RCTs comparing cispatin versus non-cisplatin treatments in the field of germ cell tumors because of ethical and medical reasons. In this case, the question of possible cisplatin-associated second cancers cannot be answered by our study and has to rely on population-based observational studies. Given the statistically nonsignificant results, we performed a posteriori power analyses [50]. We estimated the power of our meta-analysis for OR of 2.0 and 1.5 to be 99 and 75% (one-sided α of .05), respectively. Although a statistically significant increase in second cancers with cisplatin-based chemotherapy may have been missed, based on the observed incidence and relative risk, such an increase is very unlikely to change current benefit to risk balance for cisplatin. On the other hand, a sample size of 7646 will provide 80% power to rule out with 95% confidence an approximately 50% increase in the incidence of a secondary primary malignancy that occurs at a rate of 2% in the non-cisplatin group (i.e., 3% versus 2%). Because in this study, 7403 patients which is close to 7646 were included in the meta-analysis, it is reassuring that our study is not underpowered to identify small but important effects.

Another limitation is that multiple chemotherapy regimens given in the trials and various cancer types of primary cancer may limit the interpretation of our results, although we tried to perform subgroup analyses and sensitivitiy analyses. However, given that cisplatin is one of the most efficacious chemotherapeutic reagents and widely used in everyday clinical practice to treat various types of cancer, the data here are the best available from randomized trials. And the information provided here should be used for physicians and patients in the process of shared decision-making. Additionally, although an effect size was not able to be calculated for these trials, they do provide relevant data by showing that event rates for both the intervention and control groups are low and relatively equal. Friedrich [51] found that including zero total event trials in meta-analyses moves the pooled estimate of treatment effect closer to nil, but the magnitude of this increase is relatively small for RR and OR. Thus, inclusion of zero total event trials would enable the inclusion of all available randomized controlled trial data in our study, thereby providing the most generalizable estimate of treatment effect and would not significantly affect the pooled effects size.

Finally, the future reporting of long-term complications such as second cancers should be standardized. Detailed information regarding specific types of second cancers, corresponding number, location in treatment arms should be uniformly provided. Such information would be valuable when future secondary analyses of interest are performed.

## Conclusion

We found no increased risk of second cancers associated with cisplatin compared with non-cisplatin-based chemotherapy and a non-significant trend to increased risk of secondary leukemia. But the absolute risk is very low. The concern of possible risk of second cancers should not influence a decision to use an efficacious regimen containing cisplatin. This finding should be important for patient counseling and shared-decision making.

## Additional file

Additional file 1: Strategy of trials searching. (DOCX 806 kb)

## Abbreviations

CENTRAL: Cochrane Central Register of Controlled Trials
CI: Confidence interval
OR: Odds ratio
PRISMA: Preferred Reporting Items for Systematic Review and Meta-Analyses statement
RCT: Randomized controlled trials
SEER: Surveillance Epidemiology and End Results
