# Supplementary material for: Risk of second primary cancers in cancer patients treated with cisplatin: a systematic review and meta-analysis of randomized studies
Source: BMC Cancer. 2017 Dec 19;17:871. doi: 10.1186/s12885-017-3902-4 (PMC5738212; doi:10.1186/s12885-017-3902-4)
Supplement: Additional file 1: — Strategy of trials searching. (DOCX 806 kb) [file 12885_2017_3902_MOESM1_ESM.docx]

**Strategy of trials searching**

**A. Medline (PubMed) (Search date: March 28, 2016)**

#1 randomized controlled trial [pt]

#2 controlled clinical trial [pt]

#3 randomized [tiab]

#4 placebo [tiab]

#5 clinical trials as topic [mesh: noexp]

#6 randomly [tiab]

#7 trial [ti]

#8 #1 OR #2 OR #3 OR #4 OR #5 OR #6 OR #7

#9 animals [mh] NOT humans [mh]

#10 #8 NOT #9

#11 Cisplatin[mh]

#12 Cisplatin

#13 cis-Diamminedichloroplatinum (II)

#14 Platinum Diamminodichloride

#15 Diamminodichloride, Platinum

#16 cis- Platinum

#17 cis Platinum

#18 cisplatinum

#19 Dichlorodiammineplatinum

#20 cis-Diamminedichloroplatinum

#21 cis Diamminedichloroplatinum

#22 cis-Dichlorodiammineplatinum (II)

#23 Platinol

#24 Platidiam

#25 Platino

#26 NSC-119875

#27 Biocisplatinum

#28 CDDP

#29 CACP

#30 cisplatin*

#31 abiplatin

#32 neoplatin

#33 cis-DDP

#34 #11 or #12 or #13 or #14 or #15 or #16 or #17 or #18 or #19 or #20 or #21 or #22 or #23 or #24 or #25 or #26 or #27 or #28 or #29 or #30 or #31 or #32 or #33

#35 #10 and #34

**B. Embase (Ovid) (Search date: March 28, 2016)**

1 exp cisplatin derivative/ or exp cisplatin/ or cisplatin.mp.

2 cis-Diamminedichloroplatinum.mp.

3 Platinum Diamminodichloride.mp.

4 Diamminodichloride, Platinum.mp.

5 cis-Platinum.mp.

6 cis Platinum.mp.

7 cisplatinum.mp.

8 Dichlorodiammineplatinum.mp.

9 cis-Diamminedichloroplatinum.mp.

10 cis Diamminedichloroplatinum.mp.

11 cis-Dichlorodiammineplatinum.mp.

12 Platinol.mp.

13 Platidiam.mp.

14 Platinol.mp.

15 NSC-119875.mp.

16 Biocisplatinum.mp.

17 CDDP.mp.

18 CACP.mp.

19 cisplatin$.mp.

20 abiplatin.mp.

21 neoplatin.mp.

22 cis-DDP.mp.

23 1 or 2 or 3 or 4 or 5 or 6 or 7 or 8 or 9 or 10 or 11 or 12 or 13 or 14 or 15 or 16 or 17 or 18 or 19 or 20 or 21 or 22

24 random:.tw. or placebo:.mp. or double-blind:.tw.

25 23 and 24

**C. Cochrane Central Register of Controlled Trials (Search date: March 28, 2016)**

#1 Cisplatin

#2 cis-Diamminedichloroplatinum (II)

#3 Platinum Diamminodichloride

#4 Diamminodichloride, Platinum

#5 cis- Platinum

#6 cis Platinum

#7 cisplatinum

#8 Dichlorodiammineplatinum

#9 cis-Diamminedichloroplatinum

#10 cis Diamminedichloroplatinum

#11 cis-Dichlorodiammineplatinum (II)

#12 Platinol

#13 Platidiam

#14 Platino

#15 NSC-119875

#16 Biocisplatinum

#17 CDDP

#18 CACP

#19 cisplatin*

#20 abiplatin

#21 neoplatin

#22 cis-DDP

#23 MeSH descriptor: [Cisplatin] explode all trees

#24 #1 or #2 or #3 or #4 or #5 or #6 or #7 or #8 or #9 or #10 or #11 or #12 or #13 or #14 or #15 or #16 or #17 or #18 or #19 or #20 or #21 or #22 or #24

**D. ClinicalTrials.gov (Search date: March 28, 2016)**

1 Cisplatin OR cis-Diamminedichloroplatinum (II) OR (Platinum Diamminodichloride) OR (Diamminodichloride, Platinum) OR cis-Platinum OR (cis Platinum) OR cisplatinum OR Dichlorodiammineplatinum OR cis-Diamminedichloroplatinum | Studies With Results | Interventional Studies

**2** (cis Diamminedichloroplatinum) or cis-Dichlorodiammineplatinum(II) OR Platinol OR Platidiam OR Platino OR NSC-119875 OR Biocisplatinum OR CDDP OR CACP OR cisplatin* OR abiplatin OR neoplatin OR cis-DDP | Studies With Results | Interventional Studies

**3* 1 OR 2**

*This step is done by hand.

**Supplementary Table 1: Detailed information of second cancers of trials included in the study**

| Study | Cisplatin arm | | Non-cisplatin arm | |
| --- | --- | --- | --- | --- |
|  | No. of  second cancers | Detail | No. of  second cancers | Detail |
| Ajani et al^1^ | 1 | Not specified | 0 |  |
| Basu et al^2^ | 0 |  | 1 | Non-Hodgkin’s lymphoma |
| Booton et al^3^ | 0 |  | 1 | Carcinoma trachea |
| Cohen et al^4^ | 2 | Not specified | 1 | Not specified |
| Conroy et al^5^ | 7 | Lung cancer:2 ; head and neck cancer:1; hepatocarcinoma:2; renal cancer:1 ; biliary cancer:1 | 8 | Lung cancer:4; head and neck cancer:4 |
| Cortelazzo et al^6^ | 0 |  | 0 |  |
| du Bois et al^7^ | 9 | Not specified | 9 | Not specified |
| Fleming et al^8^ | 0 |  | 1 | AML |
| Fountzilas et al^9^ | 1 | Not specified | 1 | Not specified |
| Garden et al^10^ | 6 | Not specified | 1 | Not specified |
| Geyer et al^11^ | 3 | AML/MDS | 1 | MDS |
| Harari et al^12^ | 4 | Not specified | 12 | Not specified |
| Hiesiger et al^13^ | 0 |  | 1 | AML |
| Homma et al^14^ | 7 | Not specified | 7 | Not specified |
| Intragumtornchai et al^15^ | 1 | MDS | 0 |  |
| James et al^16^ | 17 | Not specified | 3 | Not specified |
| Jennings et al^17^ | 0 |  | 1 | AML |
| Nicoletto et al^18^ | 0 |  | 2 | Not specified |
| Nielsen et al^19^ | 3 | AML | 0 |  |
| Olasz et al^20^ | 1 | Larynx cancer | 1 | Oesophagus cancer |
| Sirohi et al^21^ | 3 | Not specified | 11 | Not specified |
| Sutton et al^22^ | 1 | AML/MDS | 0 |  |
| Taylor et al^23^ | 0 |  | 0 |  |
| Tropé et al^24^ | 4 | Leukemia | 2 | Leukemia |
| Tsimberidou et al^25^ | 1 | AML | 1 | Lung cancer |
| Wada et al^26^ | 3 | Not specified | 2 | Not specified |
| Wadler et al^27^ | 0 |  | 1 | Breast cancer |
| Williams et al^28^ | 1 | AML | 0 |  |

AML=Acute myeloid leukemia. MDS=Myelodysplastic syndrome.

**Supplementary Table 2: Assessment of risk of bias**

| Study | Sequence generation | Allocation concealment | Blinding of  participants and  personnel | Blinded  assessment of outcome | Incomplete outcome reporting | Selective outcome reporting |
| --- | --- | --- | --- | --- | --- | --- |
| Ajani et al^1^ | Low | Low | High | High | Low | Low |
| Basu et al^2^ | Low | Low | High | High | Low | Low |
| Booton et al^3^ | Low | Low | High | High | Low | Low |
| Cohen et al^4^ | Low | Low | High | High | Low | Low |
| Conroy et al^5^ | Low | Low | High | High | Low | Low |
| Cortelazzo et al^6^ | Low | Unclear | High | High | Unclear | Unclear |
| du Bois et al^7^ | Low | Low | High | High | Low | Low |
| Fleming et al^8^ | Low | Low | High | High | Low | Low |
| Fountzilas et al^9^ | Low | Low | High | High | Low | Low |
| Garden et al^10^ | Low | Low | High | High | Low | Unclear |
| Geyer et al^11^ | Low | Unclear | High | High | Low | Low |
| Harari et al^12^ | Low | Unclear | High | High | Low | Low |
| Hiesiger et al^13^ | Low | Low | High | High | Low | Low |
| Homma et al^14^ | Low | Low | High | High | Low | Low |
| Intragumtornchai et al^15^ | Low | Unclear | High | High | Low | Low |
| James et al^16^ | Low | Low | High | High | Low | Low |
| Jennings et al^17^ | Low | Unclear | High | High | Low | Low |
| Nicoletto et al^18^ | Low | Low | High | High | Low | Low |
| Nielsen et al^19^ | Low | Low | High | High | Low | Unclear |
| Olasz et al^20^ | High | High | High | High | Low | Unclear |
| Sirohi et al^21^ | Low | Low | High | High | Low | Low |
| Sutton et al^22^ | Low | Unclear | High | High | Low | Low |
| Taylor et al^23^ | Low | Unclear | High | High | Low | High |
| Tropé et al^24^ | Low | Low | High | High | Low | Low |
| Tsimberidou et al^25^ | Low | Unclear | High | High | Low | Low |
| Wada et al^26^ | Low | Low | High | High | Low | Low |
| Wadler et al^27^ | Low | Unclear | High | High | Low | Low |
| Williams et al^28^ | Low | Unclear | High | High | Low | Low |

Low= low risk of bias. High= high risk of bias. Unclear= unclear risk of bias.

**Supplementary Figure 1: Subgroup analysis of second cancer risk by type of control**


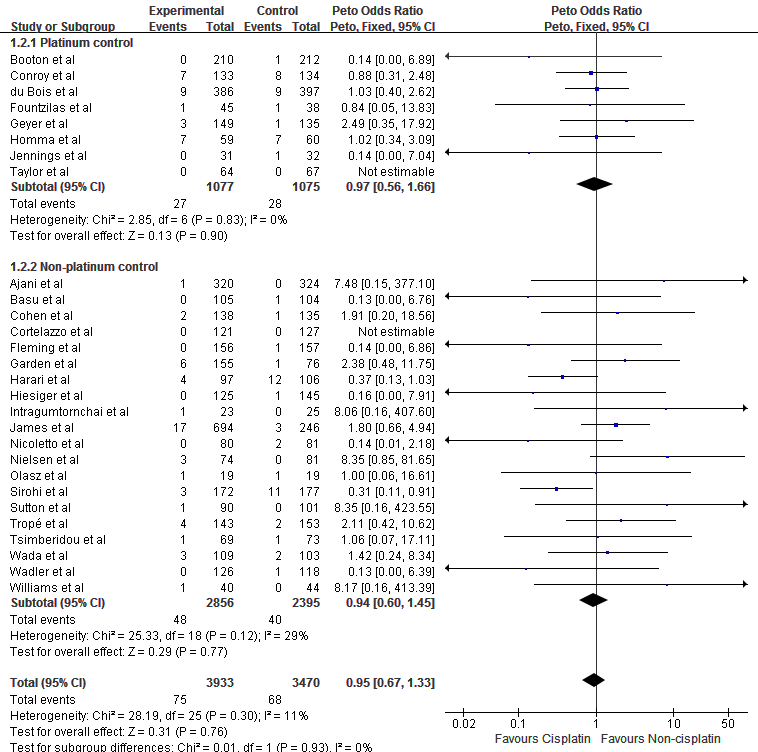

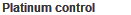

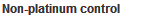


**Supplementary Figure 2: Subgroup analysis of second cancer risk by total cisplatin dose(mg/m^2^)**

**
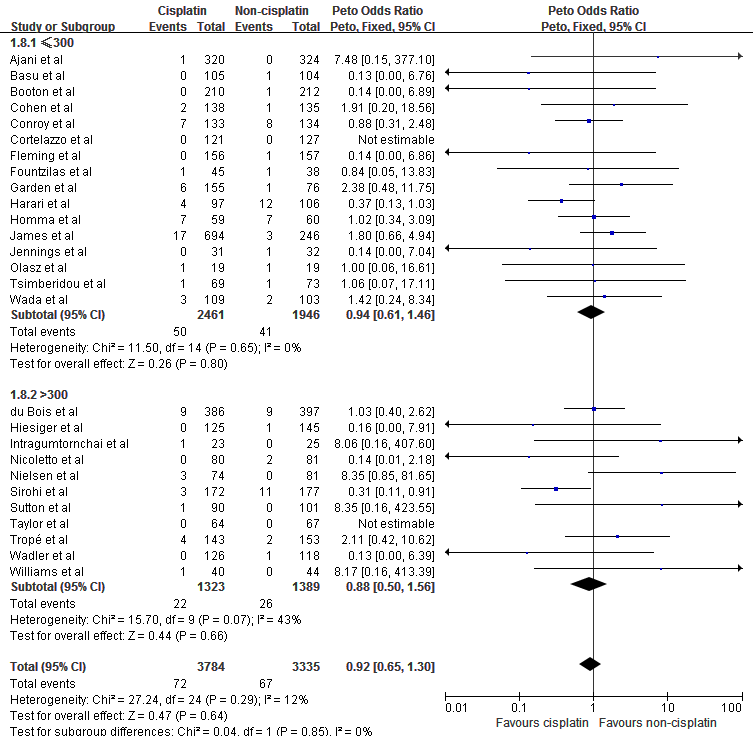
**

**Supplementary Figure 3: Subgroup analysis of second cancer risk by follow-up time (months)**


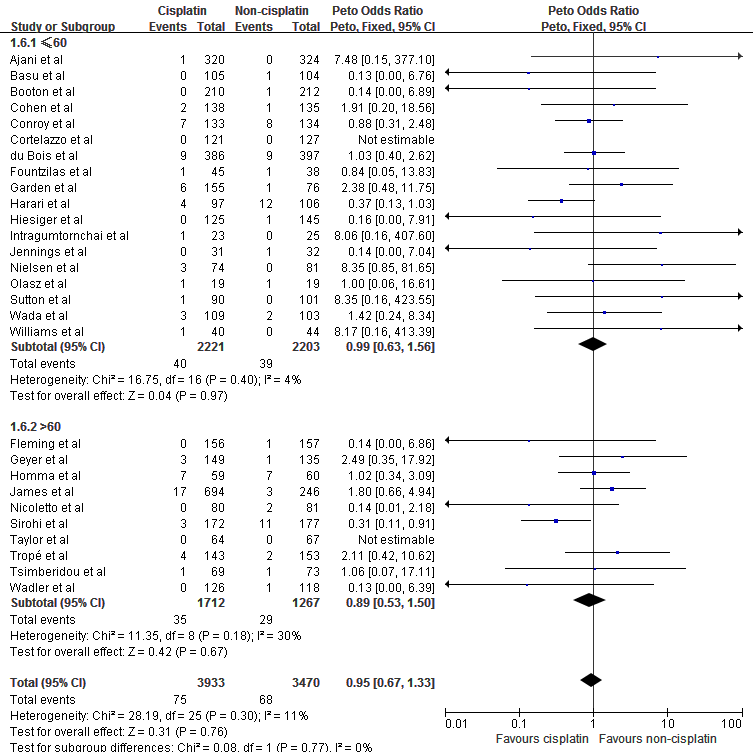

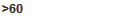

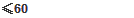


**Supplementary Figure 4: Subgroup analysis of second cancer risk by mode of treatment**


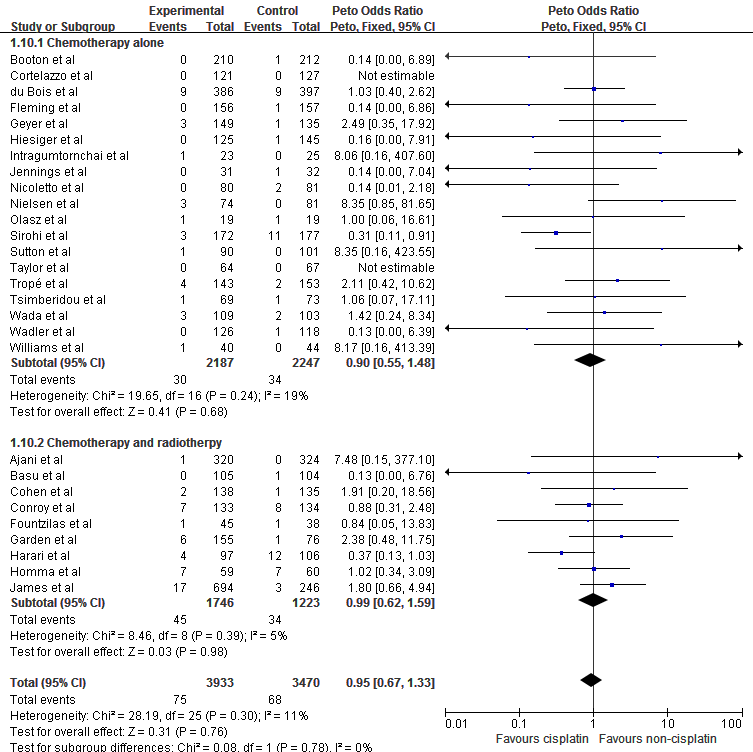

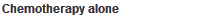

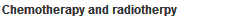


**Supplementary Figure 5: Subgroup analysis of second cancer risk by mode of comparison**


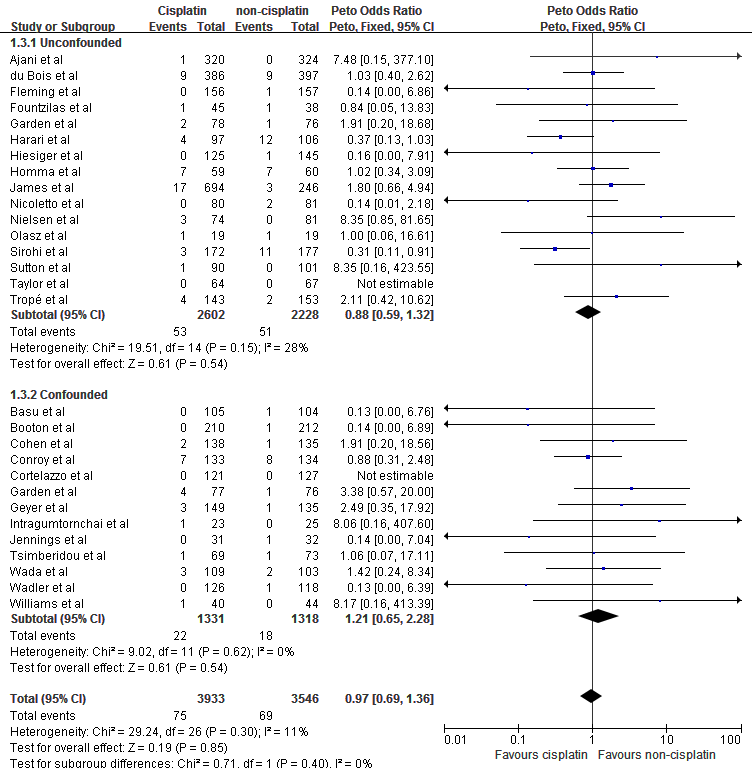

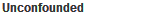

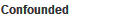


**Supplementary Figure 6: Sensitivity analysis of second cancer risk by using fixed Mantel-Hanszel statistical model**


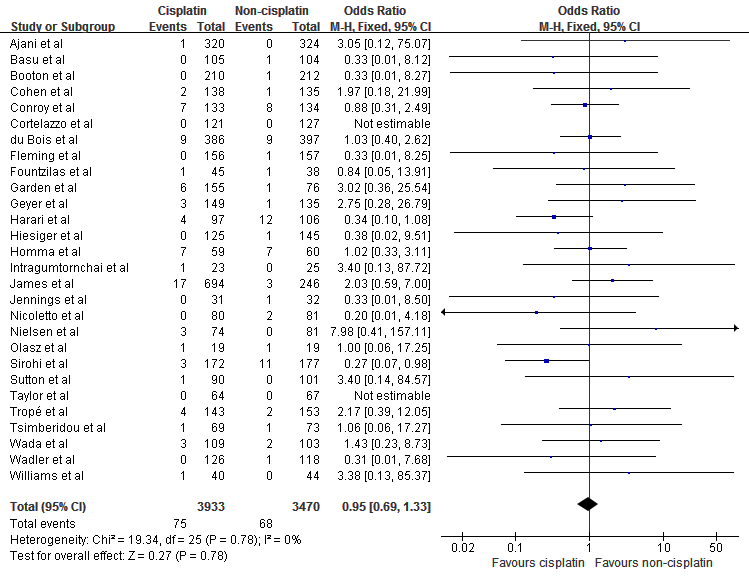

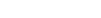


**Supplementary Figure 7: Sensitivity analysis of second cancer risk by using alternative effect measure risk ratio**


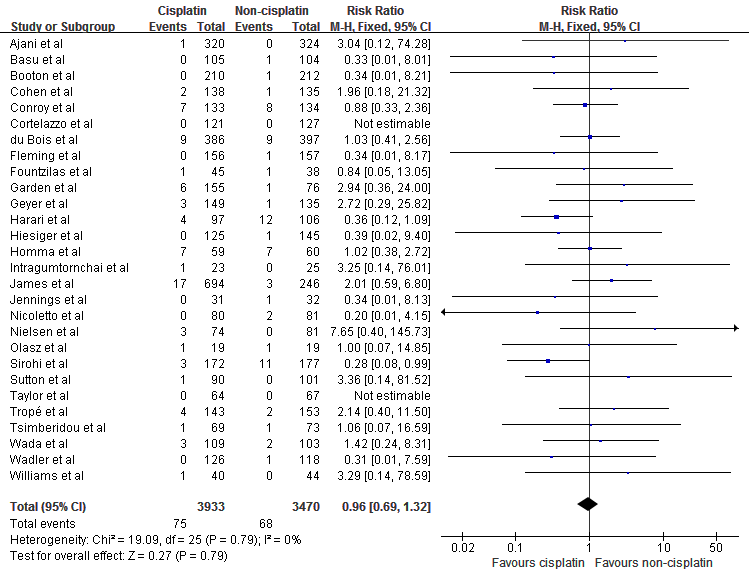

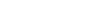


**Supplementary Figure 8: Sensitivity analysis of second cancer risk by using random effects model**


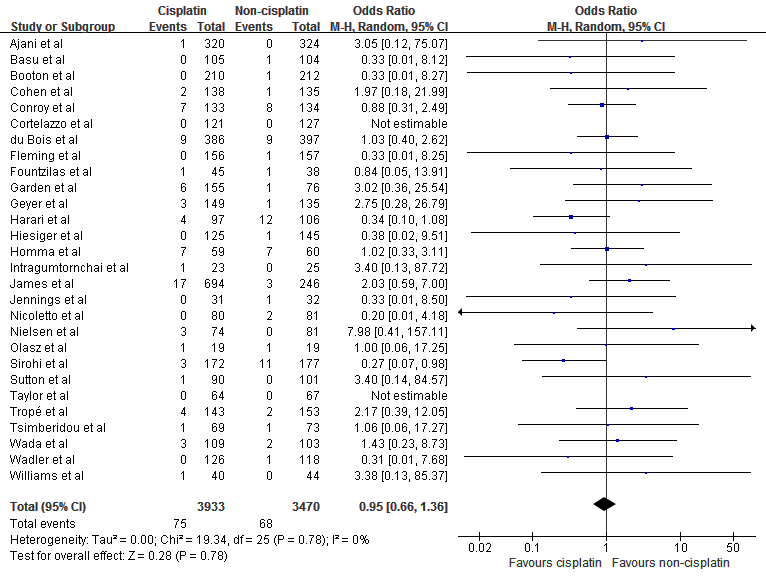

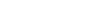


**Supplementary Figure 9: Sensitivity analysis of leukemia risk by using fixed Mantel-Hanszel statistical model**


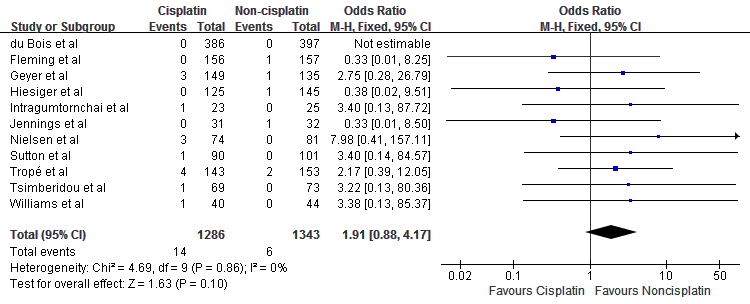

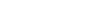


**Supplementary Figure 10: Sensitivity analysis of leukemia risk by using alternative effect measure risk ratio**


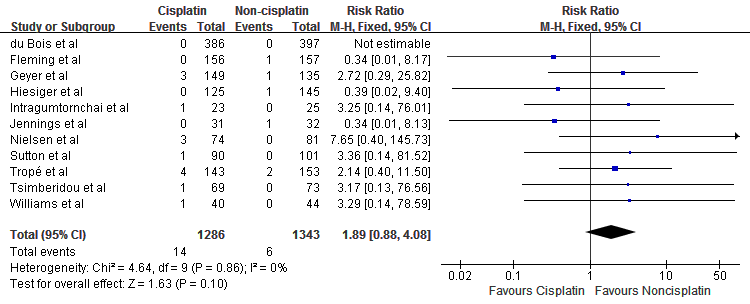

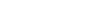


**Supplementary Figure 11: Sensitivity analysis of leukemia risk by using random effects model**


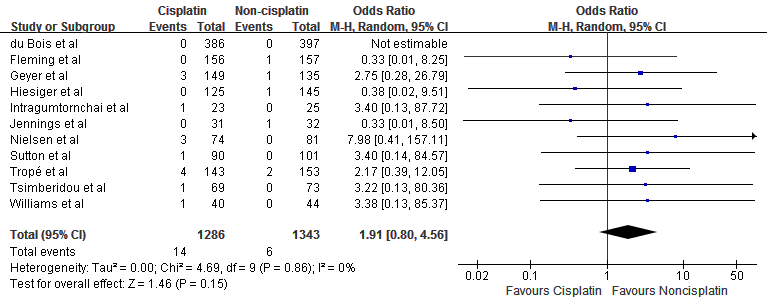

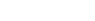


**Reference**

1. Ajani JA, Winter KA, Gunderson LL, et al. Fluorouracil, mitomycin, and radiotherapy vs fluorouracil, cisplatin, and radiotherapy for carcinoma of the anal canal: a randomized controlled trial. *Jama* 2008; 299(16): 1914-21.

2. Basu P, Jenson AB, Majhi T, et al. Phase 2 Randomized Controlled Trial of Radiation Therapy Plus Concurrent Interferon-Alpha and Retinoic Acid Versus Cisplatin for Stage III Cervical Carcinoma. *Int J Radiat Oncol Biol Phys* 2016; 94(1): 102-10.

3. Booton R, Lorigan P, Anderson H, et al. A phase III trial of docetaxel/carboplatin versus mitomycin C/ifosfamide/cisplatin (MIC) or mitomycin C/vinblastine/cisplatin (MVP) in patients with advanced non-small-cell lung cancer: a randomised multicentre trial of the British Thoracic Oncology Group (BTOG1). *Ann Oncol* 2006; 17(7): 1111-9.

4. Cohen EE, Karrison TG, Kocherginsky M, et al. Phase III randomized trial of induction chemotherapy in patients with N2 or N3 locally advanced head and neck cancer. *J Clin Oncol* 2014; 32(25): 2735-43.

5. Conroy T, Galais MP, Raoul JL, et al. Definitive chemoradiotherapy with FOLFOX versus fluorouracil and cisplatin in patients with oesophageal cancer (PRODIGE5/ACCORD17): final results of a randomised, phase 2/3 trial. *Lancet Oncol* 2014; 15(3): 305-14.

6. Cortelazzo S, Tarella C, Gianni AM, et al. Chemoimmunotherapy with R-CHOP or high dose sequential therapy with autologous stem cell transplantation (R-HDS) for high risk diffuse large B-cell lymphomas patients: Results of the randomized R-HDS0305 trial by gruppo italiano terapie innovative nei linfomi (GITIL). *Blood Conference: 54th Annual Meeting of the American Society of Hematology, ASH* 2012; 120(21).

7. du Bois A, Luck HJ, Meier W, et al. A randomized clinical trial of cisplatin/paclitaxel versus carboplatin/paclitaxel as first-line treatment of ovarian cancer. *J Natl Cancer Inst* 2003; 95(17): 1320-9.

8. Fleming GF, Filiaci VL, Bentley RC, et al. Phase III randomized trial of doxorubicin + cisplatin versus doxorubicin + 24-h paclitaxel + filgrastim in endometrial carcinoma: a Gynecologic Oncology Group study. *Ann Oncol* 2004; 15(8): 1173-8.

9. Fountzilas G, Ciuleanu E, Dafni U, et al. Concomitant radiochemotherapy vs radiotherapy alone in patients with head and neck cancer: a Hellenic Cooperative Oncology Group Phase III Study. *Med Oncol* 2004; 21(2): 95-107.

10. Garden AS, Harris J, Vokes EE, et al. Preliminary results of Radiation Therapy Oncology Group 97-03: a randomized phase ii trial of concurrent radiation and chemotherapy for advanced squamous cell carcinomas of the head and neck. *J Clin Oncol* 2004; 22(14): 2856-64.

11. Geyer JR, Sposto R, Jennings M, et al. Multiagent chemotherapy and deferred radiotherapy in infants with malignant brain tumors: a report from the Children's Cancer Group. *J Clin Oncol* 2005; 23(30): 7621-31.

12. Harari PM, Harris J, Kies MS, et al. Postoperative chemoradiotherapy and cetuximab for high-risk squamous cell carcinoma of the head and neck: Radiation Therapy Oncology Group RTOG-0234. *J Clin Oncol* 2014; 32(23): 2486-95.

13. Hieseger EM, Green SB, Shapiro WR, et al. Results of a randomized trial comparing intra-arterial cisplatin and intravenous PCNU for the treatment of primary brain tumors in adults: Brain tumor cooperative group trial 8420A. *Journal of Neuro-Oncology* 1995; 25(2): 143-54.

14. Homma A, Shirato H, Furuta Y, et al. Randomized phase II trial of concomitant chemoradiotherapy using weekly carboplatin or daily low-dose cisplatin for squamous cell carcinoma of the head and neck. *Cancer J* 2004; 10(5): 326-32.

15. Intragumtornchai T, Prayoonwiwat W, Numbenjapon T, Assawametha N, O'Charoen R, Swasdikul D. CHOP versus CHOP plus ESHAP and high-dose therapy with autologous peripheral blood progenitor cell transplantation for high-intermediate-risk and high-risk aggressive non-Hodgkin's lymphoma. *Clin Lymphoma* 2000; 1(3): 219-25.

16. James RD, Glynne-Jones R, Meadows HM, et al. Mitomycin or cisplatin chemoradiation with or without maintenance chemotherapy for treatment of squamous-cell carcinoma of the anus (ACT II): a randomised, phase 3, open-label, 2 x 2 factorial trial. *Lancet Oncol* 2013; 14(6): 516-24.

17. Jennings MT, Sposto R, Boyett JM, et al. Preradiation chemotherapy in primary high-risk brainstem tumors: phase II study CCG-9941 of the Children's Cancer Group. *J Clin Oncol* 2002; 20(16): 3431-7.

18. Nicoletto MO, Tumolo S, Sorio R, et al. Long-term survival in a randomized study of nonplatinum therapy versus platinum in advanced epithelial ovarian cancer. *Int J Gynecol Cancer* 2007; 17(5): 986-92.

19. Nielsen D, Dombernowsky P, Larsen SK, Hansen OP, Skovsgaard T. Epirubicin or epirubicin and cisplatin as first-line therapy in advanced breast cancer. A phase III study. *Cancer Chemother Pharmacol* 2000; 46(6): 459-66.

20. Olasz L, Nemeth A, Nyarady Z, Tornoczky T, Kiralyfalvi L. Results and failures with or without cisplatin containing induction chemotherapy in the treatment of squamous cell carcinoma of the head and neck. *Cancer Detect Prev* 2004; 28(1): 65-71.

21. Sirohi B, A'Hern R, Coombes G, et al. A randomised comparative trial of infusional ECisF versus conventional FEC as adjuvant chemotherapy in early breast cancer: the TRAFIC trial. *Ann Oncol* 2010; 21(8): 1623-9.

22. Sutton G, Brunetto VL, Kilgore L, et al. A phase III trial of ifosfamide with or without cisplatin in carcinosarcoma of the uterus: A Gynecologic Oncology Group Study. *Gynecol Oncol* 2000; 79(2): 147-53.

23. Taylor AE, Wiltshaw E, Gore ME, Fryatt I, Fisher C. Long-term follow-up of the first randomized study of cisplatin versus carboplatin for advanced epithelial ovarian cancer. *J Clin Oncol* 1994; 12(10): 2066-70.

24. Trope C, Andersson H, Bjorkholm E, et al. Doxorubicin-melphalan with and without cisplatin in advanced ovarian cancer--ten-year survival results from a prospective randomized study by the Swedish Cooperative Ovarian Cancer Study Group. *Acta Oncol* 1996; 35 Suppl 8: 109-18.

25. Tsimberidou AM, McLaughlin P, Younes A, et al. Fludarabine, mitoxantrone, dexamethasone (FND) compared with an alternating triple therapy (ATT) regimen in patients with stage IV indolent lymphoma. *Blood* 2002; 100(13): 4351-7.

26. Wada H, Hitomi S, Teramatsu T. Adjuvant chemotherapy after complete resection in non-small-cell lung cancer. West Japan Study Group for Lung Cancer Surgery. *J Clin Oncol* 1996; 14(4): 1048-54.

27. Wadler S, Yeap B, Vogl S, Carbone P. Randomized trial of initial therapy with melphalan versus cisplatin-based combination chemotherapy in patients with advanced ovarian carcinoma: initial and long term results--Eastern Cooperative Oncology Group Study E2878. *Cancer* 1996; 77(4): 733-42.

28. Williams CJ, Mead GM, Macbeth FR, et al. Cisplatin combination chemotherapy versus chlorambucil in advanced ovarian carcinoma: mature results of a randomized trial. *J Clin Oncol* 1985; 3(11): 1455-62.
